# Lamotrigine prophylaxis is associated with reduced aura frequency in patients with burdensome migraine aura: a retrospective observational study

**DOI:** 10.3389/fneur.2026.1863747

**Published:** 2026-07-29

**Authors:** Sena Uzun, Ulf Frejvall, Hulya Karatas, Per Petersson, Gürdal Sahin

**Affiliations:** 1 SkåNeuro Neurology Clinic, Lund, Sweden; 2 Department of Clinical Sciences of Malmö and Lund, Lund University, Lund, Sweden; 3 Integrative Neurophysiology and Neurotechnology, Department of Experimental Medical Sciences, Lund University, Lund, Sweden; 4 Institute of Neurological Sciences and Psychiatry, Hacettepe University, Ankara, Türkiye; 5 Neuroscience and Neurotechnology Center of Excellence (NÖROM), Ankara, Türkiye; 6 Department of Medical and Translational Biology, Umeå University, Umeå, Sweden

**Keywords:** antiepileptic, lamotigine, migraine aura prophylaxis, migraine with aura, preventive treatment

## Abstract

**Introduction:**

Migraine aura affects approximately one-third of migraine patients. In some individuals, aura remains highly disabling due to the lack of effective, targeted treatment options. This study evaluates the effectiveness and long-term management of lamotrigine (LTG) in a clinical Cohort specifically burdened by frequent or severe aura.

**Methods:**

A retrospective observational study was conducted at a neurology clinic in Lund, Sweden, including 81 patients treated between 2014 and 2022. The primary outcome was the proportion of patients achieving ≥50% reduction in monthly aura days (MADs) following LTG prophylaxis.

**Results:**

A total of 81 patients were included in the study. A reduction in mean MADs from 6.9 ± 7.5 days/month to 1.4 ± 3.7 was observed following LTG initiation, with 79.7% mean reduction (*p* < 0.001). The overall response rate was 85.2%. The average dose of LTG was 149.6 mg ± 70.5. Twenty-nine patients had adverse events (AE), the most common type was skin changes, and 5 dropped out of the treatment due to AEs.

**Discussion:**

The results indicate that LTG is associated with a reduction in aura frequency and is well-tolerated in patients whose migraine burden is dominated by the aura phase. LTG is a preventive option worthy of further evaluation in controlled trials.

## Introduction

Migraine aura affects one-third of migraine patients (1). Visual auras are the most common type, representing up to 98% of migraine with aura (MA) patients, though sensory, speech, and/or language, and motor symptoms may also occur (2, 3). The leading hypothesis for the pathophysiological basis of aura is cortical spreading depolarization (CSD), a slowly propagating wave of neuronal and glial depolarization followed by transient suppression of cortical activity, spreading at a rate of 3–4 mm/min (4). Neuroimaging studies, including functional MRI and, more recently, intracranial stereo EEG recordings, have indirectly and directly visualized CSD-like propagation in the visual cortex during aura episodes, providing strong support for this mechanism (5, 6). Preclinical evidence indicates that CSD can activate the trigeminovascular system and initiate migraine headache (7, 8). These observations suggest that CSD may contribute to headache initiation; however, it represents only one of several potential pathophysiological pathways. Moreover, migraine aura can also occur without headache, and most migraine attacks happen without any perceptible aura. This uncertainty has kept the role of CSD in debate and has complicated efforts to target CSD in clinical practice.

Several pharmacological agents (e.g., topiramate, valproate, lamotrigine) have demonstrated the ability to elevate the threshold for CSD or to suppress the frequency in animal models (9, 10). Their clinical efficacy in preventing aura has, however, been inconsistent, leaving a clear unmet need for targeted aura prevention and treatment (11).

The role of Calcitonin Gene-Related Peptide (CGRP) in the modulation of CSD remains a subject of debate, and consequently, the efficacy of CGRP-targeted therapies for migraine aura is not yet fully established (12–14). A case report suggested that erenumab might be effective in a patient experiencing visual aura with every attack, and recent prospective data have demonstrated that erenumab can reduce monthly aura days (MADs) (15, 16). While these findings indicate that CGRP blockers might be effective in migraine with aura, their impact is likely mediated through peripheral mechanisms and may not directly influence CSD.

In this context, lamotrigine (LTG) emerges as a particularly promising candidate. As a sodium channel blocker that reduces glutamate release and dampens cortical excitability, LTG directly targets the presumed underlying mechanisms of CSD (10). Clinically, it has demonstrated efficacy in reducing aura frequency in case series and small trials, notably without providing consistent benefit for patients with migraine without aura (MWoA) (17–21).

In this study, we selected a distinct group of migraine with aura patients characterized by a high burden of migraine aura. In these individuals, aura remains highly disabling, partly because its role in migraine pathophysiology is still unclear, and partly because treatments that specifically target the presumed underlying mechanism are lacking.

The aim of this study is to evaluate the clinical effectiveness of LTG as a targeted preventive therapy in this cohort. Furthermore, we aim to provide real-world insights into long-term management.

## Materials and methods

This retrospective cohort study was conducted at SkåNeuro Neurology Clinic in Lund, Sweden. The data were drawn from medical records of migraine patients who were treated with LTG between January 2014 and December 2022.

### Participants

Patients were identified through a retrospective review of medical records. Patients were included if they met International Classification of Headache Disorders, 3rd edition (ICHD-3) criteria for migraine with aura and were prescribed LTG by their treating neurologist based on a documented clinical history of burdensome migraine aura (22). Burdensome aura was defined as high frequency or severity of aura symptoms that served as the primary clinical indication for initiating preventive therapy, or when the aura phase was perceived by the patient as more disabling or prominent than the headache phase. Patients were excluded if they had concomitant epilepsy, a diagnosed mood disorder, or if baseline aura data were missing from the clinical record.

Since this is a retrospective study, the initiation of LTG was decided by the treating physicians according to their clinical assessment and the needs of each patient. Laboratory assessments, including serum electrolytes, were performed approximately every other year during long-term follow-up.

### Variables

Demographic characteristics, migraine features, aura types, the presence of extreme photosensitivity, aura frequency, and concomitant medications were extracted from patients’ medical records. Extreme photosensitivity was defined as persistent, abnormal photophobia extending into the interictal period, to the extent that some patients reported an inability to function without sunglasses even indoors.

The primary outcome was the proportion of patients achieving at least a 50% reduction in MADs from baseline to the end of the study period. MADs were calculated as the total number of days per month on which aura symptoms occurred. Baseline MADs were determined from clinical documentation covering the most recent 1–3 months period prior to LTG initiation, depending on the availability and completeness of headache diaries or clinical notes. Post-treatment MADs were calculated from the last available headache diary entry in the medical files prior to the end of the study period (December 2022), which varied by patient depending on their final documented assessment and reported as treatment duration. Clinical response was categorized as follows: non-responders—for a reduction of less than 50% and super-responders—for a reduction exceeding 75% (23). Additionally, the adverse events (AEs) rate was evaluated.

LTG dosing was individualized by the treating neurologists based on clinical response. Treatment was initiated at a dose of 25 mg, with gradual titration in 25 mg increments every 2 weeks until reaching 100 mg. Upon reaching this dose, a clinical evaluation was conducted. In cases of unsatisfactory effect, the dose could be increased by 25–50 mg increments every 3–6 months until an effective dose was achieved. Based on our clinical experience and evidence from epilepsy treatment, 400 mg was established as the maximum dose.

### Statistical analysis

Descriptive statistics were performed to summarize the data initially. For categorical data, counts and percentages were used, and the numerical data results are presented as mean ± standard deviation (SD) or median (interquartile range) when appropriate. Shapiro–Wilk’s test was used to assess data normality. As the data were not normally distributed, the Wilcoxon signed-rank test was used to compare the monthly aura days (baseline vs. post-treatment). Differences in the time to reach the maximum dose between patients with and without AEs were evaluated using the Mann–Whitney U test. The Kruskal-Wallis test was used to evaluate the effectiveness of different aura types. Analyses were performed using RStudio with statistical significance set at *p* < 0.05, two-tailed.

### Ethical approval

The study was approved by the Swedish Ethical Review Authority with a diary number of 2022–00542-01, and all enrolled patients provided written informed consent. The study was conducted in accordance with the Declaration of Helsinki. Additionally, the present study adheres to the guidelines outlined in the Strengthening the Reporting of Observational Studies in Epidemiology (STROBE) Statement.

## Results

A total of 92 patients were examined; 4 were excluded due to concomitant epilepsy, 1 declined to provide informed consent, and 5 had not started the medication despite the neurologist’s recommendation, 1 was excluded due to missing baseline aura days data. Thus, in the final analysis, 81 patients were included.

The most common aura type was visual aura, reported in 98.7% of patients, followed by sensory aura (45.6%) and speech and/or language disturbances (32.0%). Additionally, 9.8% of patients exhibited extreme photosensitivity. In total, 62 patients had a family history of migraine, with missing data for 7 patients. Baseline MADs were 6.9 days ± 7.5 in a month prior to the treatment. Patient characteristics are shown in detail in Table 1.

**Table 1:** Characteristics of study population (*n* = 81).

Variable	Value
Female, *n* (%)	55 (67.9)
Age, mean ± SD (range)	49.8 ± 16.3 years (22–87)
Family history, *n* (%)	62
Migraine without aura	52
Migraine with aura	10
Baseline monthly aura days (MADs), ± SD, (range)	6.9 ± 7.5 days/month (0,3–30)
Aura type, *n* (%)	
Visual	80 (98.7)
Sensory	37 (45.6)
Speech and/or language	26 (32.0)
Previous preventive treatment against migraine, *n* (%)	33 (40.7)
Beta blockers	30
Tricyclic antidepressants	6
Angiotensin receptor blocker	4
Antiepileptic drugs (other than lamotrigine)	2
Calcium channel blockers	1
Botulinum toxin A	1
Concomitant treatments, *n* (%)	14 (17.2)
Beta blockers	1
Botulinum toxin A	9
Anti-CGRP mAb	3
Angiotensin receptor blocker	1

In this cohort, no patients presented with migraine aura without headache. Additionally, 10 (12.3%) patients reported occasional migraine without aura attacks. LTG was the first preventive therapy for 45 patients, with missing data for 3 patients. Although 59.3% of the patients received LTG as their first preventive therapy, they were not newly diagnosed with migraine with aura. As a tertiary referral center, most patients had previously been evaluated elsewhere but had not received prior preventive treatment.

The average maintenance dose of LTG was 149.6 mg ± 70.5, and the median time to reach maintenance dose was 11 months (IQR: 2–29). Median duration of LTG treatment during the observation period was 44 months (IQR: 19–92).

**Figure 1:**
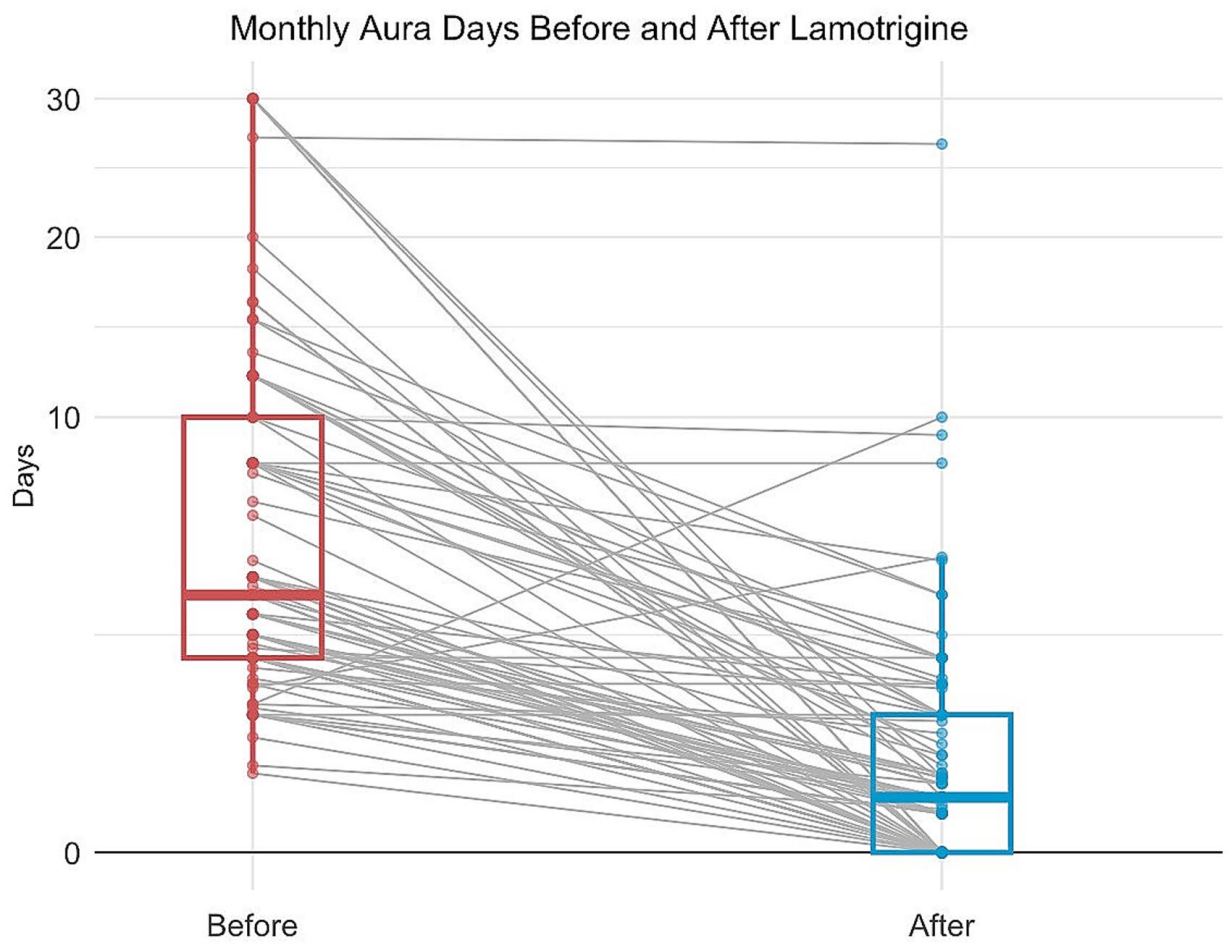
Change in monthly aura days (MADs) before and after lamotrigine treatment. Each pair of dots represents a different patient (*n* = 81). The red boxplot shows MADs before treatment, and the blue boxplot shows MADs after a median of 11 months of lamotrigine therapy. The mean MADs decreased from 6.9 days ± 7.5 to 1.4 days ± 3.7, representing a 79.7% reduction rate (*p* < 0.001).

**Figure 2:**
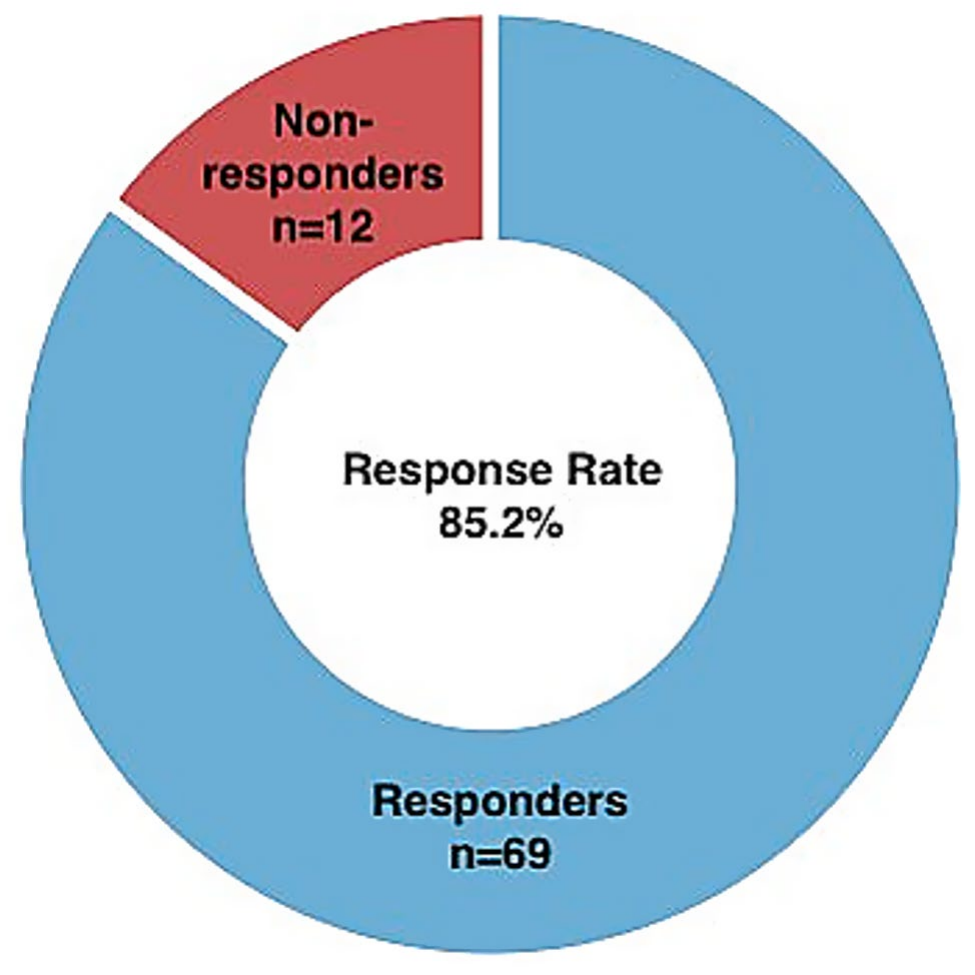
Proportion of responders and non-responders among study participants. The donut illustrates the overall response rate, with 85.2% (*n* = 69) classified as responders and 14.8% (*n* = 12) as non-responders.

A reduction in mean MADs from 6.9 ± 7.5 to 1.4 ± 3.7 was observed following LTG initiation, with median baseline MADs of 3.5 (IQR: 2.0–10.0) declining to 0.2 (IQR: 0.0–1.0) days/month post-treatment, representing a 79.7% mean reduction (*p* < 0.001) (Figure 1). Overall, 69 patients (85.2%) responded to LTG treatment, among them 65 patients were super-responders. While 12 (14.8%) of the patients were non-responders (Figure 2). In total 45 patients were treatment-naïve, with missing data for 3 patients. Among treatment-naïve patients, 6 (13.3%) were non-responders, while 6 (18.2%) of the previously treated patients (*n* = 33) were classified as non-responders, showing no statistically significant difference between these two groups (*p* = 0.24). Furthermore, patients receiving LTG as monotherapy (*n* = 67) were analyzed separately, and the treatment effectiveness was maintained in this subgroup (*p* < 0.001).

Fourteen patients (17%) continued to have migraine without aura attacks on LTG treatment and have been treated with other different preventive medications (Table 1). Patients receiving LTG as monotherapy (*n* = 67) demonstrated greater treatment effectiveness compared to those receiving concomitant preventive medications (*n* = 14). In the monotherapy group, baseline MADs were 7.0 days/month ± 7.7, declining to 0.8 days/month ± 2.0 post-treatment, with a response rate of 92.5% (*p* < 0.001). In contrast, patients receiving concomitant preventive treatment had baseline MADs of 6.9 days/month ± 7.1, declining to 4.7 days/month ± 7.2, with a response rate of 50.0% (*p* = 0.041). No significant difference in baseline aura burden was observed between those two groups (*p* = 0.99), suggesting that the marked difference in treatment response was not attributable to baseline severity differences. Additionally, no significant difference in effectiveness was found between different aura types (*p* = 0.97).

In total, 29 patients (35.8%) experienced AEs. The most common AE was skin-related changes, reported in 14 patients. These included maculopapular rash, mild erythema, dry skin, hair loss, and photosensitivity. Cognitive impairments, including impaired concentration and amnesia, and diffuse, non-specific symptoms such as dizziness and fatigue, were observed in 11 patients. One patient exhibited psychological symptoms characterized by visual hallucinations of insects. Electrolyte disturbances were identified in 3 patients. Among the 29 patients who experienced AEs, 24 recovered fully, while 5 discontinued the treatment due to AEs: one due to Stevens-Johnson syndrome, one due to minor pruritus of the hands and fingers, two due to urticaria, and one due to fatigue.

**Figure 3:**
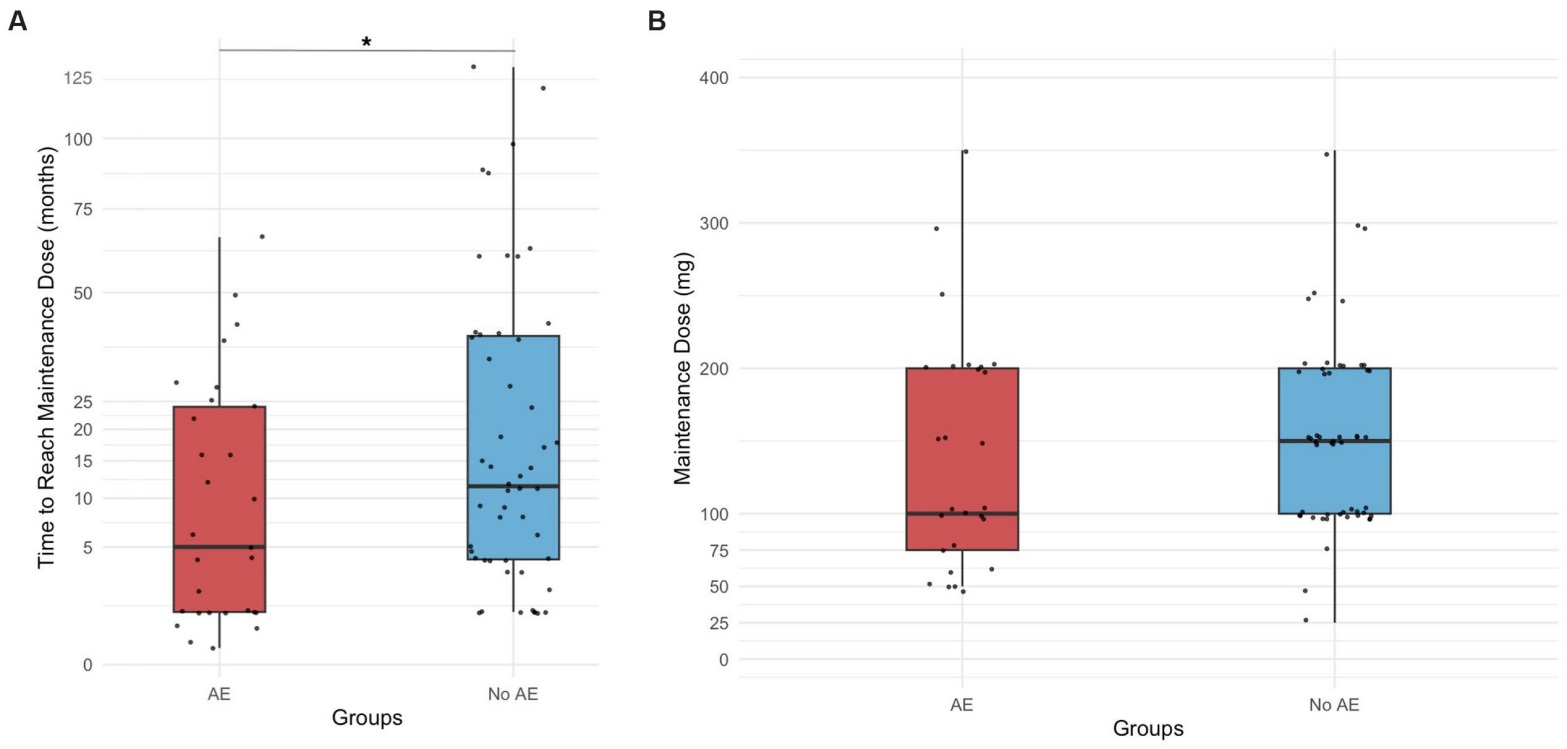
**(A)** Time to reach maintenance dose in patients with and without adverse events (AEs). The median time to reach the maintenance dose was significantly shorter in patients who experienced AEs (5.0 months) compared to those who did not (11.5 months) (*p = 0*.03). **(B)** Maintenance dose in patients with and without adverse events (AEs). The median maintenance dose was 100 mg in the AE group and 150 mg in the non-AE group. No statistically significant association was observed between maintenance dose and AE occurrence (*p* = 0.27).

Among patients who experienced AEs, the median time to reach the maintenance dose was 5.0 months, compared to 11.5 months in those who did not experience AEs. A statistically significant inverse association was observed between the occurrence of AEs and the time to reach the maintenance dose (*p* = 0.03), meaning that reaching the maintenance dose more quickly corresponded with experiencing more AEs (Figure 3A). The maintenance dose was not found to be significantly different between the groups, although the median LTG dose was 100 mg in the AE group and 150 mg in the non-AE group (*p* = 0.27) (Figure 3B).

## Discussion

This observational study found that LTG was associated with a significant and profound reduction in MADs among patients with burdensome migraine aura. Notably, we observed a high response rate of 85.2%, accompanied by a favorable safety and tolerability profile. The literature presents some conflicting findings regarding the efficacy of LTG; however growing evidence in recent years increasingly supports its therapeutic potential. Early open-label studies demonstrated that LTG can reduce both the frequency and duration of aura and, in some cases, migraine attacks (17–19, 24). For example, D’Andrea et al. reported a reduction in aura attacks; however the attack frequency remained unchanged in 5 patients with MWoA. LTG has also been suggested as a potentially beneficial option for persistent visual aura (25). More recently, an open-label, prospective study in migraine patients with comorbid anxiety and depression reported that LTG may reduce attack frequency even in MWoA patients, possibly due to LTG’s mood-stabilizing effects (26).

In contrast, placebo-controlled trials have reported limited benefits. Steiner et al. found no significant difference between LTG and placebo in reducing migraine frequency. Similarly, Gupta et al. observed only modest efficacy of LTG compared to topiramate (20, 21). However, a large retrospective study by Smeralda et al. found LTG to be as effective as topiramate for aura reduction, with superior tolerability (27). This discrepancy between retrospective studies, including ours, and controlled trials is possibly due to different cohort selections. Our results, along with others, suggest that choosing patients with burdensome migraine aura, as in this study’s cohort, should be considered a key criterion for patient selection in LTG treatment.

In our cohort, patients also demonstrated improvements in migraine headache frequency, which may reflect the consistent association between aura and headache in this cohort. Only 14 patients (17.2%) required additional preventive treatments beyond LTG, underscoring its potential as a monotherapy in appropriately selected cohorts.

In this cohort, where aura represented the predominant or most disabling feature of migraine attacks, it is plausible that CSD is not only present but may be central to the pathophysiology. This may explain the high response rate of LTG observed in this cohort, as its proposed ability to raise the threshold for CSD initiation and suppress cortical hyper excitability may potentially target the underlying mechanism (10). Thus, it supports the notion that preventing CSD could play a role in mitigating both aura and the subsequent headache in patients whose attacks are CSD-driven or CSD-dependent.

### Evidence suggesting a therapeutic potential of LTG in aura prevention

Various antiepileptic drugs are employed in migraine prophylaxis due to their modulatory effects on neuronal excitability. However, their long-term utility is frequently constrained by unfavorable side effect profiles, including cognitive impairment, weight fluctuations, and fatigue (28, 29). This has spurred interest in developing more targeted therapies, particularly those capable of suppressing CSD. Despite promising preclinical data, efforts to translate CSD-specific inhibitors into clinical practice have been unsuccessful. Candidates such as picotamide (a dual thromboxane inhibitor) and tonabersat (a gap junction modulator) demonstrated efficacy in animal models but failed to show consistent clinical benefits in migraine prevention (30–32).

LTG has emerged as a potential alternative due to its distinct mechanisms of action. Preclinical studies indicate that LTG reduces neuronal hyperexcitability and suppresses CSD propagation in animal models (10). These effects are primarily mediated through its inhibition of voltage-gated sodium and calcium channels (33). Unlike other anticonvulsants, LTG also modulates the TWIK-related spinal cord potassium (TRESK) channel—a mitochondrial-regulated potassium channel, which has been suggested to play a role in migraine pathogenesis (34–36).

Collectively, these findings position LTG as a plausible candidate for targeted aura prevention. Its ability to attenuate CSD either directly (via ion channel modulation) or indirectly (through TRESK-mediated effects on neuronal stability) distinguishes it from other prophylactic options. Clinically, this profile may be particularly relevant for this cohort, who often experience limited relief from standard therapies. Future studies should explore optimized dosing regimens and patient stratification to evaluate LTG’s efficacy in patients with disabling migraine aura, potentially bridging the gap between preclinical promise and clinical application.

### Adverse events profile

In the current study, 29 patients experienced AEs; however, only 5 (6.1%) patients discontinued the treatment because of AEs. In contrast, a placebo-controlled study initiating LTG at a full dose of 200 mg reported a 39% discontinuation rate due to AEs (20). Most previous studies followed a relatively fixed titration protocol—starting at 25 mg and increasing by 25 mg every week or every other week (17, 18, 26, 27).

Our data indicate that slower titration was associated with a lower incidence of AEs (Figure 3A), meaning that patients who reached the maintenance dose more quickly were more likely to experience AEs. This observation suggests slower titration could be explored as a strategy to reduce AEs in future studies. Furthermore, the median LTG dose was not significantly different between the groups (100 mg in patients who experienced AEs and 150 mg in those who did not), indicating that the occurrence of AEs is unlikely to be dose-dependent but rather more related to the titration rate.

### Demographic features of the study cohort

In this cohort, 67.9% of patients were women. This is consistent with epidemiological data, which estimate that approximately 66% of patients with MA are women, with a lifetime prevalence of 5% (1, 37). More recent studies have reported higher rates, with women comprising around 72% of MA patients, and up to 90% in specific clinical cohorts (3, 38). While the clinical presentation of migraine varies, it is estimated that 10% of patients experience more than 75% of their attacks, a high-burden phenotype that was represented in our study population (39). Given the limited sample size in the present study, interpretation of these findings remains challenging, highlighting the need for larger epidemiological investigations within MA.

The most common aura type was visual aura (98.7%), which aligns with existing literature. Recent studies have similarly demonstrated that visual aura is the most prevalent form, occurring in up to 99% of cases, and that it is almost always present when multiple aura types are reported by the same patient (2, 3).

### Limitations and strengths

This study has inherent limitations due to its retrospective design, single-center setting, and absence of a control group. Although the retrospective design limits causal inference, the consistent documentation of patient outcomes over a long follow-up period provides valuable real-world evidence. Moreover, the baseline period was not predefined but reconstructed retrospectively from available clinical records, which spanned approximately 1 to 3 months before LTG initiation and varied slightly between patients. Due to the variable follow-up duration among patients (median 44 months, IQR 19–92), the time window for post-treatment assessment varied by patient, which may introduce variability in the comparability of treatment outcomes across the cohort. In addition, data on aura duration were not consistently documented, preventing evaluation of pre-to-post treatment changes.

The subjective definition of burdensome aura and individualized dosing strategy present limitations to the reproducibility of our findings. Dose titrations and final maintenance dosages were determined by treating clinicians based on patient response and the emergence of AEs; hence, a standardized dosing algorithm was not utilized. This variability reflects real-world clinical practice but may complicate the direct replication of our treatment transition in other settings.

The main focus of this study was migraine aura, which resulted in limited data on headache features such as frequency and severity. To partially address this, we reviewed concomitant medications used for headache management.

To the best of our knowledge, this study has the largest patient group and longest follow-up period currently available in the literature concerning LTG use for burdensome migraine aura. Consequently, these findings offer a valuable contribution to the existing but limited literature on the subject.

### Future directions

This cohort of patients provides a unique opportunity to explore the role of CSD in migraine. To further strengthen this theoretical framework, future research in neuroimaging of the brain during migraine aura, either through human provocation studies or spontaneous attacks, is needed. Furthermore, randomized placebo-controlled trials are warranted to validate the efficacy of LTG. Such studies could provide a more detailed understanding of LTG titration, its efficacy in aura duration and intensity, as well as its effect on migraine headache.

A recent statement advocating higher standards in migraine prevention encourages clinicians and researchers to raise expectations for treatment outcomes in migraine care (40). These improved outcomes should also include the prevention and treatment of aura. In this context, we would like to emphasize once again the importance of effective aura management, particularly for patients experiencing highly disabling auras or those whose professions demand precise sensory functioning, such as pilots or drivers. Addressing aura symptoms is essential for improving patients’ quality of life.

In conclusion, LTG may be an effective prophylactic treatment for reducing aura burden in patients with burdensome migraine aura, providing a high response rate and a favorable safety profile when titrated gradually. The marked improvement in MADs and high responder rate underscore LTG’s potential as a targeted monotherapy for this cohort. Given the central role of CSD in aura, LTG’s mechanisms could provide a strong pathophysiology-based rationale for this preventive strategy. These findings support more personalized treatment approaches and suggest the need for further controlled studies to validate LTG’s role in managing migraine aura.

## Data Availability

The raw data supporting the conclusions of this article will be made available by the authors, without undue reservation.
